# Assisted hatching of vitrified-warmed blastocysts prior to embryo transfer does not improve pregnancy outcomes

**DOI:** 10.1186/s13048-020-00692-x

**Published:** 2020-08-04

**Authors:** Charis Ng, Marta Wais, Taryn Nichols, Sarah Garrow, Julius Hreinsson, Zhong-Cheng Luo, Crystal Chan

**Affiliations:** 1 Faculty of Medicine, University of Toronto, Toronto, Ontario Canada; 2 Mount Sinai Fertility, Toronto, Ontario Canada; 3 Lunenfeld-Tanenbaum Research Institute, Department of Obstetrics and Gynecology, Mount Sinai Hospital, University of Toronto, 250 Dundas Street West, 7th Floor, Toronto, Ontario M5G 1X5 Canada

**Keywords:** Assisted hatching, Zona breach, Zona thinning, Vitrified-warmed blastocyst

## Abstract

**Objective:**

This study aims to determine the impact of assisted hatching (AH) on pregnancy outcomes in vitrified-warmed blastocyst transfers, and evaluate if embryo expansion or morphology influences outcomes.

**Methods:**

A retrospective cohort study was performed including vitrified-warmed blastocyst transfers at our clinic between 2013 and 2017. Of the 2165 embryo transfers, 1986 underwent laser AH and 179 were non-assisted hatched (NAH). The primary outcome was live birth. Secondary outcomes included conception, implantation, clinical pregnancy, clinical pregnancy loss, and monozygotic twinning (MZT).

**Results:**

AH and NAH groups had similar rates of conception (38.7% vs 42.1%), implantation (26.2% vs 27.3%), clinical pregnancy (29.1% vs 30.3%), clinical pregnancy loss (24.0% vs 17.8%), live birth (19.9% vs 20.5%), and MZT (2.08% vs 2.86%). Five pairs of dichorionic/diamniotic twins resulted from single embryo transfers. AH of embryos with expansion grades ≤3 was associated with lower rates of conception (32.5% vs 44.3%%, *p* < 0.05) and clinical pregnancy (24.0% vs 32.8%, *p* < 0.05).

**Conclusion:**

AH prior to transfer of vitrified-warmed blastocysts was not associated with improved pregnancy outcomes. The identification of dichorionic/diamniotic twins from single blastocyst transfers challenges the previously held notion that dichorionic/diamniotic MZTs can only occur from division prior to the blastocyst stage. Prospective studies are needed to validate the novel finding of lower rates of conception and clinical pregnancy after AH in embryos with lower expansion grade.

## Introduction

The zona pellucida (ZP) is a glycoprotein layer surrounding the oocyte that is required for sperm-oocyte binding, prevention of polyspermy, and oviductal transportation [1]. After fertilization and blastocyst formation, in order for implantation to occur, the embryo must first shed its ZP in a process called “hatching” [2]. Physiologically, it is believed that trophectoderm cells secrete lysins that chemically thin the ZP, and the embryo undergoes repeated expansion-contraction cycles that contribute to the breaching of the ZP and eventual hatching [3, 4].

In IVF, it has been suggested that prolonged embryo culture may impair the embryo’s ability to hatch and ultimately implant [5]. In some patient populations, including those with advanced reproductive age, repeated implantation failure, or cryopreserved embryos, a hardening of the ZP has been suggested [2, 6, 7]. To facilitate hatching of in vitro cultured embryos, assisted hatching (AH) was proposed by Cohen in 1988 [8]. AH refers to any laboratory technique that artificially thins or breaches the ZP of an embryo prior to transfer. Current and historical techniques include mechanical dissection with a glass micro-needle, chemical drilling with Tyrode’s solution, enzymatic thinning, and laser thinning [9]. The use of this technique in clinical practice has become commonplace, and it is estimated that AH was performed in 44.8% of IVF cycles in the United States between the years 2000 and 2010 [10].

However, three decades on, there remains poor clinical consensus on whether AH improves implantation and pregnancy outcomes. A Cochrane Review demonstrated significantly improved clinical pregnancy rates with AH, but this association just reached statistical significance [11]. However, guidelines from the National Institute for Clinical Excellence (NICE), the American Society for Reproductive Medicine (ASRM), and the Society for Assisted Reproductive Technology (SART) recommend against the routine use of AH for patients undergoing IVF [10, 12]. Additionally, previous studies have suggested that AH may be associated with higher rates of monozygotic twinning, resulting in more high risk pregnancies and adverse obstetrical or neonatal outcomes [13, 14].

The available literature on the use of AH prior to embryo transfers is primarily focused on cleavage stage embryos. However, as extended culture, blastocyst transfer, and vitrification have superseded cleavage stage transfers, the literature requires updating. In addition, many of the studies on frozen-thawed blastocysts have focused on specific patient populations such as those with repeated implantation failure, which limits generalizability of findings [15–23]. To date, there have been no studies evaluating the impact of laser AH exclusively on vitrified-warmed blastocyst transfers in an unselected IVF population. It is also poorly understood whether or not AH outcomes vary depending on the morphologic characteristics of the blastocyst embryo or its ZP. Inferring from the evidence that cleavage stage embryos with reduced ZP thickness have higher implantation potential, a previous study attempted to determine if the effectiveness of AH depends on ZP thickness [24, 25]. The results demonstrated that AH improved the implantation rate for cleavage stage embryos with ZP thickness ≥ 15 μm. However, to our knowledge no previous studies have investigated the impact of blastocyst expansion or morphology on pregnancy outcomes after AH.

The objectives of this study are to assess whether laser AH enhances the implantation ability, rate of clinical pregnancy, or rate of live birth for vitrified-warmed blastocysts in an unselected population and to identify embryological factors that may mediate this effect.

## Materials and methods

### Patients

We conducted a retrospective cohort study including all vitrified-warmed blastocyst embryo transfers (FET) performed at our fertility clinic during the five-year period between January 1, 2013 and December 31, 2017. Institutional research ethics board approval was obtained from Mount Sinai Hospital, University of Toronto. Informed consent was waived since the study did not involve individual contact with patients. During the study reference period, a total of 3206 FET cycles were started. Embryo transfers that occurred after pre-implantation genetic testing (PGT) were excluded from this study because, inherent to the PGT technique, all embryos underwent laser breaching of the ZP prior to embryo biopsy. Transfers that involved ≥2 embryos with discordant use of AH (eg. one embryo hatched and not the other) were also excluded since it was not possible to determine which outcome was associated with which embryo. FET cycles that were cancelled before embryo transfer for various reasons (eg. failure of embryo to survive thaw, patient-initiated cancellation) were also excluded.

### Vitrification and thaw methods

Blastocysts were vitrified using ICE vitrification/warming media (Innovative Cryo Enterprise, New Jersey, US) and HSV vitrification straws (Fertitech, Saint-Laurent, Quebec, Canada). Briefly, the blastocysts were equilibrated at room temperature in solutions containing increasing concentrations of cryoprotectants with a 1–2 min exposure to the final solution before loading the embryo onto the HSV straw. Finally, the carrier straw was sealed and plunged into the liquid nitrogen.

At the time of warming, the straw was opened using a cutter while the embryo remained below the level of liquid nitrogen. The inner straw containing the embryo was rapidly plunged into the first warming solution and the cryoprotectants were removed through serial dilution by transferring the embryo through the remaining solutions at room temperature. The embryo was then returned to culture before transfer.

### Assisted hatching procedures

In total, 2165 embryo transfers were included. The decision on whether or not to perform AH was based on physician discretion and/or patient preference, given the lack of evidence on the efficacy of the intervention. All patients receiving this treatment provided written informed consent prior to AH. The AH group included 1986 embryo transfers, while the comparison group (NAH, non-assisted hatched) included 179 embryo transfers.

Laser AH was performed immediately after thawing, with the blastocyst placed in Total Global HP media. Either ZILOS-tkZona Infrared Laser Optical System (version 5.12.0.31735, Hamilton Thorne Biosciences, Beverly, USA) or OCTAX LaserShot&EyeWare MX (v.1.7, Build 437, OCTAX Microscience GmbH, Bruckberg, Germany) were used at a power of 100% and pulse of 500 μs. Prior to July 2014, no specifications were made regarding the diameter of ZP breaching, however after that time, breached holes were standardized to ≥30 μm.

### Endometrial preparation for FET

Endometrial preparation for FET involved either a natural or hormone replacement protocol, as per physician and/or patient preference. The natural cycle protocol was as follows: cycle monitoring with trans-vaginal ultrasound and serial serum estradiol, LH, and progesterone measurements until an endometrial stripe thickness (EST) ≥8 mm with a dominant follicle of ≥1.8 cm is attained. This is followed by administration of recombinant human chorionic gonadotropin (hCG) (Ovidrel, EMD Serono, Canada) 250 μg subcutaneously to trigger luteinization. Two hundred milligram of supplemental micronized progesterone (Prometrium, Merck, Canada) is given twice daily per vagina starting the day after trigger. The hormone replacement protocol was as follows: micronized 17 beta-estradiol (Estrace, Acerus Pharmaceuticals, Canada) 4 mg twice daily per vagina starting on day 2 of the menstrual cycle and continued for a minimum of 12 days until an EST ≥8 mm is attained, then micronized progesterone (Prometrium, Merck, Canada) 200 mg three times daily per vagina is started. With either protocol, FET was performed after five full days of progesterone treatment, on the morning of the sixth day (“P + 5”). Embryo transfers were completed using a double lumen embryo transfer catheter (Cook, Canada) under transabdominal ultrasound guidance. Note that there are some studies that have shown benefit with transvaginal ultrasound guidance [26, 27], but at this point the standard approach at our facility and many others is transabdominal.

### Outcomes and statistical analysis

The primary outcome was live birth. Secondary outcomes included conception, implantation, clinical pregnancy, clinical pregnancy loss, and monozygotic twinning rates. Conception was defined as a serum quantitative βhCG level > 5 mIU measured 9 days after FET. Implantation rate was defined as the number of gestational sacs detected on trans-vaginal ultrasound 6–7 weeks post-transfer divided by the number of embryos transferred. Clinical pregnancy was defined as the presence of one or more gestational sacs detected on trans-vaginal ultrasound 6–7 weeks post-transfer. Clinical pregnancy loss was defined as loss of a documented intrauterine pregnancy prior to 20 weeks gestation age excluding therapeutic abortions. Monozygotic twinning was defined as the presence of 1) more fetal heart beats than the number of embryos transferred, or 2) more fetal heart beats than the number of gestational sacs seen on trans-vaginal ultrasound at 6-7 weeks post-transfer [28].

Additionally, a subgroup analysis was conducted on all FET cycles where a single embryo was transferred in order to determine if blastocyst expansion grade or morphology influenced AH outcomes. The first variable assessed was expansion grade which was derived from the laboratory-assigned Gardner score [29]. A second variable explored was the morphology of the blastocyst, which was measured using the modified Society for Assisted Reproductive Technology (SART) embryo scoring system as reported by Heitmann et al. [30]. A SART grade of “good” was assigned to embryos with an inner cell mass (ICM) grade of A and trophectoderm (TE) grade of A or B (AA or AB blastocysts). A SART grade of “fair” was assigned for an ICM grade of B and TE grade of A, B or C (BB, BC, or BA blastocysts). A SART grade of “poor” was assigned for any embryos with an ICM grade of C (CC or CB blastocysts) (Table 1).

**Table 1:** Modified Society for Assisted Reproductive Technology (SART) embryo scoring system

*Modified SART score*	*Gardner score*
*Good*	AA, AB
*Fair*	BA, BB, BC
*Poor*	CB, CC

The differences in categorical variables were compared by chi-square tests, and in continuous variables by t tests. Generalized estimated equations (GEE) were applied to assess the adjusted risk ratios of the outcomes comparing AH vs NAH accounting for co-variables and cluster-level effects (multiple rounds of embryo transfer in the same patient). All data analyses were conducted using Statistical Analysis System (SAS), version 9.2 (SAS Institute, NC). A *p*-value < 0.05 was considered statistically significant.

## Results

The clinical characteristics of patients in the AH and NAH groups are presented in Table 2, and no clinically significant differences were found. There was a statistically significant difference in mean age at the time of oocyte retrieval (33.8 ± 3.9 years and 33.1 ± 3.7 years in the AH and NAH groups, respectively), but this difference is likely attributable to sample size and may not be clinically significant due to the very slight difference.

**Table 2:** Baseline clinical characteristics of study patients

	*Assisted Hatched (n = 1986)*	*Non-Assisted Hatched (n = 179)*	*P-value*ª
**Mean maternal age at retrieval**	33.8 ± 3.9	33.1 ± 3.7	0.03
**Mean maternal age at transfer**	35.3 ± 4.0	34.6 ± 4.0	0.02
**Cause of infertility**			
Ovulatory disorder	14.2% (283)	17.9% (32)	0.18
Tubal factor	30.5% (606)	32.3% (58)	0.54
Endometriosis	7.0% (139)	7.3% (13)	0.89
Male Factor	34.5% (686)	34.1% (62)	0.97
Other	30.4% (604)	24.7% (45)	0.16
**Endometrial preparation protocol**			
Natural Cycle	11.2% (223)	13.7% (25)	0.31
Hormone Replacement Therapy	88.8% (1764)	86.0% (154)	0.31
**Number of embryos transferred per cycle**			
1	74.1% (1474)	69.8% (125)	0.24
2	25.5% (506)	30.2% (54)	0.16
3	0.3% (6)	0.0% (0)	0.91
4	0.1% (2)	0.0% (0)	0.61
**Previous transfers (Fresh & Vitrified-warmed)**			
0	14.4% (287)	13.7% (25)	0.80
1	34.8% (692)	34.6% (63)	0.97
2	22.5% (444)	17.2% (37)	0.08
3	13.5% (269)	13.7% (23)	0.24
> 4	15.0% (296)	16.8% (31)	0.40

ª*P* values are for comparisons of continuous (t test) or categorical (chi-square test) variables between the two groups

The clinical outcomes and adjusted risk ratios (from GEE models) comparing AH vs NAH are shown in Table 3. No statistically significant differences were noted between groups with regard to conception (38.7% vs 42.1%, *p* = 0.35), implantation (26.2% vs 27.3%, *p* = 0.76), clinical pregnancy (29.1% vs 30.3%, *p* = 0.34), clinical pregnancy loss (24.0% vs 17.8%, *p* = 0.34), and live birth (19.9% vs 20.5%, *p* = 0.46) rates. Monozygotic twinning rates also appeared to be comparable between the two groups (2.1% vs 2.9%, *p* = 0.55). However, it is important to note that the low number of events with this outcome (*n* = 8 and 1, respectively) significantly limits data interpretation.

**Table 3:** Comparisons of pregnancy outcomes for vitrified-warmed blastocyst transfers

	*Assisted Hatched (n = 1986)*	*Non-Assisted Hatched* *(n = 179)*	*aRR*ª *(95% CI)*	*P-value*
*Conception Rate*	38.7% (768)	42.1% (75)	0.90 (0.59–1.36)	0.35
*Implantation Rate*	26.2%	27.3%	–	0.76
*Clinical Pregnancy Rate*	29.1% (576)	30.3% (54)	0.93 (0.54–1.60)	0.34
*Clinical Pregnancy Loss Rate*	24.0% (121)	17.8% (8)	1.39 (0.34–5.66)	0.34
*Live Birth Rate*	19.9% (383)	20.5% (35)	0.98 (0.49–1.96)	0.46
*Monozygotic Twinning Rate*	2.1% (8)	2.9% (1)	0.49 (0.004–55.76)	0.55

ªThe adjusted RRs were from generalized estimated equations (GEE) adjusted for patient clinical characteristics and woman cluster-level (multiple rounds of embryo transfer in the same patient) variation

A subgroup analysis of single embryo transfers (*n* = 1599) was conducted to determine if embryo morphology or expansion grade influence outcomes after AH. Based on frequency distribution, embryos were grouped into those with an expansion grade ≤ 3 and those with an expansion grade of 4. For embryos with an expansion grade of 4, AH did not have a significant impact on pregnancy outcomes (Table 4). In embryos with an expansion grade ≤ 3, AH was associated with a statistically significant decrease in conception (32.5% vs 44.3%, *p* = 0.01) and clinical pregnancy (24.0% vs 32.8%, *p* = 0.02) rates (Table 5). No statistically significant differences were seen with regards to implantation (24.4% vs 34.4%, *p* = 0.12), clinical pregnancy loss (17.9% vs 11.8%, *p* = 1.00) and live birth (17.6% vs 22.4%, *p* = 0.17) rates.

**Table 4:** Comparisons of pregnancy outcomes for single embryo transfers with expansion grade of 4

	*Assisted Hatched (n = 680)*	*Not Assisted Hatched (n = 62)*	*RR (95% CI)*	*P-value*
*Conception Rate*	39.3% (267)	37.1% (23)	1.06 (0.76–1.48)	0.79
*Implantation Rate*	31.0%	25.8%	–	0.38
*Clinical Pregnancy Rate*	30.0% (204)	25.8% (16)	1.16 (0.75–1.80)	0.56
*Clinical Pregnancy Loss Rate*	28.8% (53)	15.4% (2)	1.87 (0.51–6.84)	0.52
*Live Birth Rate*	18.9% (125)	15.3% (9)	1.24 (0.67–2.31)	0.60

**Table 5:** Comparison of pregnancy outcomes for single embryo transfers with expansion grade of ≤3

	*Assisted Hatched (n = 755)*	*Not Assisted Hatched (n = 61)*	*aRR*ª *(95% CI)*	*P-value*
*Conception Rate*	32.5% (245)	44.3% (27)	0.43 (0.23–0.84)	0.01
*Implantation Rate*	24.4%	34.4%	–	0.12
*Clinical Pregnancy Rate*	24.0% (181)	32.8% (20)	0.38 (0.17–0.87)	0.02
*Clinical Pregnancy Loss Rate*	17.9% (29)	11.8% (2)	2.54 (0.15–42.00)	1.00
*Live Birth Rate*	17.6% (130)	22.4% (13)	0.47 (0.16–1.40)	0.17

ªThe adjusted RRs were from generalized estimated equations (GEE) adjusted for patient clinical characteristics and woman cluster-level (multiple rounds of embryo transfer in the same patient) variation

Analyses investigating the role of morphology on AH outcomes demonstrated no significant impact for embryos graded “poor”, “fair”, or “good” according to the modified SART embryo scoring system (Table 6). Specifically, when comparing AH to NAH, embryos graded “poor” or “fair” had a conception rate of 35.4% vs 38.8% (*p* = 0.52), implantation rate of 27.2% vs 28.2% (*p* = 0.83), clinical pregnancy rate of 26.5% vs 27.2% (*p* = 0.91), clinical pregnancy loss rate of 17.9% vs 14.3% (*p* = 1.00), and live birth rate of 17.6% vs 16.3% (*p* = 0.89), respectively. Similarly, no statistically significant differences were seen between AH and NAH groups among embryos graded “good” with regards to conception (36.9% vs 50.0%, *p* = 0.34), implantation (29.2% vs 40.0%, *p* = 0.36), clinical pregnancy (28.4% vs 40.0%, *p* = 0.31), clinical pregnancy loss (25.1% vs 13.0%, *p* = 0.31), and live birth (21.1% vs 31.6%, *p* = 0.26) rates in AH vs NAH embryo transfers.

**Table 6:** Comparison of pregnancy outcomes for single embryo transfers based on modified SART scores

	*Modified SART Score*	*Assisted Hatched*	*Non-Assisted Hatched*	*RR (95% CI)*	*P-value*
*Conception Rate*	Good	36.9% (100)	50.0% (10)	0.74 (0.46–1.18)	0.34
	Fair/Poor	35.4% (412)	38.8% (40)	0.91 (0.71–1.18)	0.52
*Implantation Rate*	Good	29.2%	40.0%	–	0.36
	Fair/Poor	27.2%	28.2%	–	0.83
*Clinical Pregnancy Rate*	Good	28.4% (77)	40.0% (8)	0.71 (0.40–1.25)	0.31
	Fair/Poor	26.5% (308)	27.2% (28)	0.97 (0.70–1.35)	0.91
*Clinical Pregnancy Loss Rate*	Good	25.1% (70)	13.0% (3)	1.92 (0.66–5.63)	0.31
	Fair/Poor	17.9% (12)	14.3% (1)	1.25 (0.19–8.27)	1.00
*Live Birth Rate*	Good	21.1% (55)	31.6% (6)	0.67 (0.33–1.35)	0.26
	Fair/Poor	17.6% (200)	16.3% (16)	1.08 (0.68–1.72)	0.89

## Discussion

To our knowledge, this is the largest study of its kind to evaluate the impact of AH on vitrified-warmed blastocyst transfers in an unselected IVF population. The results suggest that AH does not improve pregnancy outcomes. Additionally, the present study demonstrates that AH has no significant impact regardless of embryo morphology. AH does not appear to have an impact on outcomes in embryos with an expansion grade of 4; however, statistically significant decreases in conception and clinical pregnancy rates were seen in embryos that were less expanded.

Previous studies on the outcomes of AH on vitrified-warmed embryo transfers have reported conflicting results. In a randomized study, Wan et al. employed quarter ZP opening by laser AH on the day of transfer in patients undergoing transfer of blastocyst embryos that developed from vitrified-warmed low-grade cleavage stage embryos [21]. They reported statistically significant increases in clinical pregnancy (51.0% vs 35.3%, *p* = 0.034) and implantation rates (34.2% vs 23.6%, *p* = 0.021) with AH, but not live birth rates (40.6% vs 28.4%, *p* > 0.05) [21]. Another randomized study by Zhang et al. studied different sizes of ZP thinning using laser AH on slow frozen-thawed cleavage stage embryos. They reported an improvement in pregnancy (defined by two consecutive tests showing elevated beta-hCG) and implantation rates with 80 μm thinning of the ZP compared to no hatching (40.3% vs 23.5%, *p* = 0.007; 21.5% vs 7.5%, *p* = 0.03, respectively) [18]. They also reported increased implantation rates with 80 μm thinning compared to 40 μm thinning (21.5% vs.9.4%, *p* = 0.024) [18]. A retrospective study by Hiraoka et al. on laser AH of good quality blastocysts developed from slow frozen-thawed cleavage stage embryos in patients with recurrent implantation failure reported a significant decrease in delivery rates for non-hatched groups compared to those with 40 μm ZP opening (13% vs 38%, *p* < 0.05) and 50% ZP opening (13% vs 65%, *p* < 0.01) [17].

Other studies that have found more similar results to the present study include a prospective RCT conducted by Debrock et al. This study looked at the impact of modified quarter laser-assisted ZP thinning compared to no hatching in Day 1, 2, 3, and 5 embryos that had either undergone slow freezing or vitrification [25]. The majority of all embryos included in the study were day 3 embryos undergoing slow freeze or vitrification (52.2 and 15.8%, respectively). Only 1.0% and 10.8% of all embryos included in the study were slow frozen or vitrified day 5 embryos, respectively. Overall, they found no significant differences between the quarter AH and control groups with respect to implantation (12.9% vs 15.4%, *p* = 0.35), clinical pregnancy (24.8% vs 23.5%, *p* = 0.75), and live birth (15.3% vs 17.5%, *p* = 0.54) rates.

The differences in findings between the existing studies and our own may be attributed to variations in study population, embryo cryopreservation technique, and AH timing/technique. Over the past decade, our laboratory and many others have shifted from cleavage stage to extended blastocyst culture, and from slow-freezing to vitrification for embryo cryopreservation [31–33]. Our current practice is to vitrify, thaw, hatch, and transfer embryos at the blastocyst stage. This is in contrast to older studies on AH that either transferred cleavage stage embryos after cryopreservation or used slow-freezing techniques. There is some evidence that embryo stage may influence the outcomes of AH. Tannus et al. demonstrated in a retrospective study on fresh embryo transfers in women aged 40 and above that AH was associated with reduced live birth rate with cleavage stage embryos but not with blastocysts [34]. In addition, with current methods of vitrification, thawed blastocysts may not benefit from AH as much as they did with past freeze-thaw techniques. Indeed, there is evidence in bovine embryos that the ZP displays different ultra-structural alterations after slow freeze compared to vitrification [35].

The other difference between our study and others is our standardization of laser AH to a ZP opening size of ≥30 μm. Literature on the topic of assisted hatching has been completely heterogeneous with respect to the size of ZP opening, ranging all the way to complete mechanical removal of the zona pellucida [36]. Further studies are required to determine whether an ideal ZP opening size exists for vitrified-warmed blastocyst embryos that balances safety and efficacy.

No previous studies in the vitrified-warmed blastocyst population have observed sufficient monozygotic twinning events to comment on potential associations with AH. Our study, similarly, had too few events to draw a definitive conclusion. However, we were able to specifically document this event, which has been lacking in many previous studies [16]. Interestingly, our study identified 5 sets of diamniotic/dichorionic (di/di) twins in the AH cohort that resulted from single embryo transfers. Barring a second spontaneous conception in the same cycle, this could only have occurred through post-transfer monozygotic splitting of the transferred blastocyst [37]. The most interesting implication of our finding of di/di monozygotic twinning events after blastocyst transfer is that it challenges previously held notion that monozygotic di/di twinning can only occur prior to the blastocyst stage [38]. Recently published studies not looking specifically at AH have also challenged this empirical belief, including Kyono et al. who reported at least 12 cases of monozygotic di/di twinning occurring after day 4 [38]. Additionally, to date, monozygotic splitting of cultured embryos has never been witnessed in the ART laboratory even with extended culture. It is possible our study underestimates the actual incidence of monozygotic twinning and missed cases that could not be captured by our definition (more fetal heart beats than number of embryos transferred or more fetal heart beats than gestational sacs on ultrasound). For example, di/di monozygotic twins resulting from double embryo transfers in which one embryo splits and the other fails to implant would be unavoidably missed.

Although several teams have studied AH in select blastocyst subgroups based on embryo grading, to the best of our knowledge none have previously explored the effects of expansion grade or embryo morphology on AH outcomes [17, 19–21]. Our study found that AH has no significant effect on outcomes regardless of embryo morphology, however expansion grade may be an important variable to consider. Physiologically, as an embryo undergoes expansion, its ZP undergoes a natural process of thinning [39]. Thus, in theory, embryos with lower expansion grades should benefit more from AH than fully expanded embryos [2]. Our study found no differences in outcomes with AH in embryos that have an expansion grade of 4. However, contrary to our hypothesis, a detrimental effect of AH was seen in conception and clinical pregnancy rates for embryos with lower expansion grades. A conceivable mechanism by which AH may negatively affect embryo implantation is through damage from laser and physical manipulation of the embryo. It is possible that less expanded embryos may be more vulnerable to damage from manipulation. Although more studies are needed to validate this finding, our data cautions against the routine use of AH for vitrified-warmed blastocyst embryo transfers, particularly with less expanded blastocysts.

Given the retrospective and non-randomized nature of this study, several limitations exist including unmeasurable bias and missing data. Specifically, decisions to provide AH or NAH were based on the experience of the treating physician, with some physicians routinely performing AH as their standard of care and others routinely performing transfers without AH. However, it is reassuring that clinical characteristics were similar in AH and NAH patients in our study cohort, indicating the lack of selection bias or confounding by indication. Additionally, we were able to stratify embryos by morphology and expansion grade, however were unable to directly assess the impact of ZP thickness on AH outcomes due to the fact that our lab does not routinely record ZP thickness before laser AH. Also, our protocol for AH is standardized to a ZP opening of ≥30 μm. This enhances the comparability between the AH and NAH groups, but also removes the possibility to assess the effect of opening size. Future large prospective studies are required to further explore how the thickness of the ZP and size of the laser breach may impact outcomes.

## Conclusions

Laser AH of vitrified-warmed blastocyst embryos prior to transfer has no effect on IVF outcomes, including the rates of conception, implantation, clinical pregnancy, clinical pregnancy loss, live birth, and monozygotic twinning, regardless of blastocyst morphology. It may be associated with poorer conception and clinical pregnancy outcomes in embryos with lower expansion grades, however further studies in other cohorts are required to validate this new finding and elucidate the etiology of this phenomenon. Our data does not support the routine use of AH in this population to improve IVF outcomes. The identification of several events of di/di twinning from single blastocyst transfers challenges the previously held notion that monozygotic splitting occurs only prior to the blastocyst stage.

## Data Availability

The datasets generated and/or analysed during the current study are not publicly available due to ethical restrictions.
